# GEP100/Arf6 Is Required for Epidermal Growth Factor-Induced ERK/Rac1 Signaling and Cell Migration in Human Hepatoma HepG2 Cells

**DOI:** 10.1371/journal.pone.0038777

**Published:** 2012-06-11

**Authors:** ZhenZhen Hu, Jun Du, Ling Yang, YiChao Zhu, Yu Yang, DaTong Zheng, Akimasa Someya, Luo Gu, Xiang Lu

**Affiliations:** 1 Department of Physiology, Nanjing Medical University, Nanjing, China; 2 Department of Pediatrics, The Second Affiliated Hospital of Nanjing Medical University, Nanjing, China; 3 State Key Laboratory of Reproductive Medicine, Nanjing Medical University, Nanjing, China; 4 Department of Cardiology, The Third Affiliated Hospital of Suzhou University, Suzhou, China; 5 Department of Host Defense and Biochemical Research, Juntendo University School of Medicine, Tokyo, Japan; 6 Cancer Center, Nanjing Medical University, Nanjing, China; 7 Department of Geriatrics, The Second Affiliated Hospital of Nanjing Medical University, Nanjing, China; Hungarian Academy of Sciences, Hungary

## Abstract

**Background:**

Epidermal growth factor (EGF) signaling is implicated in the invasion and metastasis of hepatoma cells. However, the signaling pathways for EGF-induced motility of hepatoma cells remain undefined.

**Methodology/Principal Findings:**

We found that EGF dose-dependently stimulated the migration of human hepatoma cells HepG2, with the maximal effect at 10 ng/mL. Additionally, EGF increased Arf6 activity, and ectopic expression of Arf6 T27N, a dominant negative Arf6 mutant, largely abolish EGF-induced cell migration. Blocking GEP100 with GEP100 siRNA or GEP100-△PH, a pleckstrin homology (PH) domain deletion mutant of GEP100, blocked EGF-induced Arf6 activity and cell migration. EGF also increased ERK and Rac1 activity. Ectopic expression GEP100 siRNA, GEP100-△PH, or Arf6-T27N suppressed EGF-induced ERK and Rac1 activity. Furthermore, blocking ERK signaling with its inhibitor U0126 remarkably inhibited both EGF-induced Rac1 activation as well as cell migration, and ectopic expression of inactive mutant form of Rac1 (Rac1-T17N) also largely abolished EGF-induced cell migration.

**Conclusions/Significance:**

Taken together, this study highlights the function of the PH domain of GEP100 and its regulated Arf6/ERK/Rac1 signaling cascade in EGF-induced hepatoma cell migration. These findings could provide a rationale for designing new therapy based on inhibition of hepatoma metastasis.

## Introduction

Epidermal growth factor (EGF) has a profound effect on the differentiation of specific cells *in vivo*, and has been shown to be a potent mitogenic factor for a variety of cultured cells [1]. It is noteworthy that EGF produced by tumor-associated macrophages also acts as a chemoattractant in promoting motility of various types of human cancer cells [2], [3]. Specific inhibition of EGF receptors (EGFR) abolishes cytoskeleton remodeling and migration of cancer cells in response to EGF [4], [5]. However, the molecular mechanisms underlying the effect of EGF/EGFR on tumor cell migration are not completely understood to date.

EGFR is made up of an extracellular ligand-binding domain, a short hydrophobic transmembrane domain and a cytoplasmic tyrosine kinase-containing domain. Binding of ligands to the extracellular domain of EGFR induces the formation of receptor homo- or heterodimers, and subsequent activation of the intrinsic tyrosine kinase domain. These phosphorylated residues serve as docking sites for proteins containing Src homology 2 (SH2) and phosphotyrosine binding (PTB) domains, such as Grb2, Crk and the intercellular kinase Src, and leads to activation of different signaling molecules that transmit the signal in the cell [6]. Indeed, activation of EGFR by EGF has been found to induce tumorigenesis through upregulation of signaling pathways, including PI3K/Akt, STAT, Ras/Raf/MAPK [7], [8] and members of Rho GTPase family such as Rac1 [9].

GEP100, one of the guanine nucleotide exchanging factors (GEFs) for Arf6, has been implicated in EGF signaling and cancer invasion. GEP100 is expressed in most of primary breast ductal carcinomas, and is preferentially co-expressed with EGFR in malignant cases [10]. It has been shown that GEP100 interacts specifically with EGFR and plays a pivotal role in promoting tumor invasion both *in vitro* and *in vivo* [10]. In a study on human hepatoma HepG2 cells, GEP100 interacts directly with α-catenin and regulates actin cytoskeleton remodeling and cell adhesion [11]. Inhibition of GEP100 by siRNA was also reported to enhance cell attachment and spreading on fibronectin-coated substrates [12]. GEP100 contains a *Sec7* domain, an incomplete IQ-motif, and a pleckstrin homology (PH) domain. The PH domain of GEP100 differs greatly from that of other Arf GEFs in regions involved in phospholipid binding [13]. In fact, the PH domain of GEP100 was identified to bind directly to Tyr1068/1086-phosphorylated EGFR and was required for EGF-stimulated Arf6 activation in MDA-MB-231 breast cancer cells [14]. Because Arf6 has been identified to play an important role in cancer cell migration [15] and the PH domain of GEP100 links EGFR signaling to Arf6 activation, it is worthwhile to explore whether the PH domain of GEP100 is involved in EGF-induced Arf6 signaling pathway and cancer cell migration ability. Arf6 has been identified as a potent modulator of extracellular-signal-regulated kinase (ERK) and Rac1 activity [16], [17]. Rac1, one of the best-characterized member of small GTPases family, was reported to be associated with lamellipodial dynamics and chemotactic migration [18]. Although a cross-talk between signaling from Arf6, ERK and Rac1 may occur in different cellular processes, the precise molecular mechanisms implicating GEP100 in cancer cell motility have not yet been unraveled. In the present study, we investigated the signaling mechanisms underlying the effect of GEP100, especially the function of its PH domain, on hepatoma cell migration. Our results demonstrate that EGF stimulates hepatoma HepG2 cell migration through GEP100-dependent activation of the Arf6/ERK/Rac1 signaling pathway.

## Results

### EGF Stimulates Migration of HepG2 Cells in vitro

**Figure 1:**
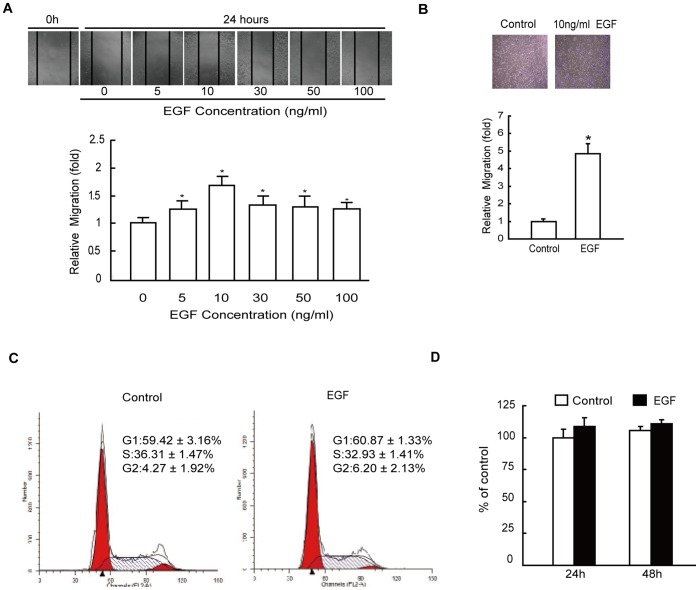
Effect of EGF on migration of HepG2 cells. (A) Relative cell migration rate was determined using wound closure assay in HepG2 cells incubated in the absence or presence of 5, 10, 30, 50, and 100 ng/mL EGF for 24 h. (B) The cell migration was assessed by the Transwell migration assay in HepG2 cells incubated in the absence (control) or presence of 10 ng/mL EGF for 8 h. (C) HepG2 cells were cultured in the absence (control) or presence of 10 ng/mL EGF for 24 h and cell cycle was analyzed by flow cytometry. (D) HepG2 cells were cultured in the absence (control) or presence of 10 ng/mL EGF for 24 or 48 h and cell proliferation was analyzed by MTT assays. Each value represents the mean ± SD of 5 independent determinations. *: *P*<0.05, referring to the difference between cells treated with and without EGF.

To assess the effect of EGF on cancer cell migration, human hepatoma HepG2 cells were treated with various concentrations of EGF, and the migration rate of cells was measured by wound closure assay as described in ";Materials and Methods";. Similar to the findings of Price *et al*. [19], we found that 5 ng/mL EGF caused an increase in cell migration over untreated cells. Maximal increase in cell migration was observed with 10 ng/mL EGF, which induced an approximately 1.7-fold increase in cell migration over untreated cells and tapered off with further increase in the dose of EGF up to 100 ng/mL (Fig. 1A). Cell migration was also assessed by Transwell migration assays. HepG2 cells were treated with 10 ng/mL EGF, which increased the number of migrated cells by 5 folds over that of controls (Fig.1B). To determine whether EGF-induced cell migration was associated with increased cell proliferation, we treated HepG2 cells with 10 ng/mL EGF for 24 h, and cellular proliferation was examined by cell cycle analysis. The results revealed that the percentages of cells in the S and G2 phase were not altered significantly in EGF treated cells compared to the control cells (Fig. 1C). Our MTT assays additionally showed that treatment with 10 ng/ml EGF for 24 and 48 h did not noticeably increase the proliferation of HepG2 cells (Fig. 1D).

### GEP100/Arf6 Regulates EGF-induced Cell Migration *in vitro*

**Figure 2:**
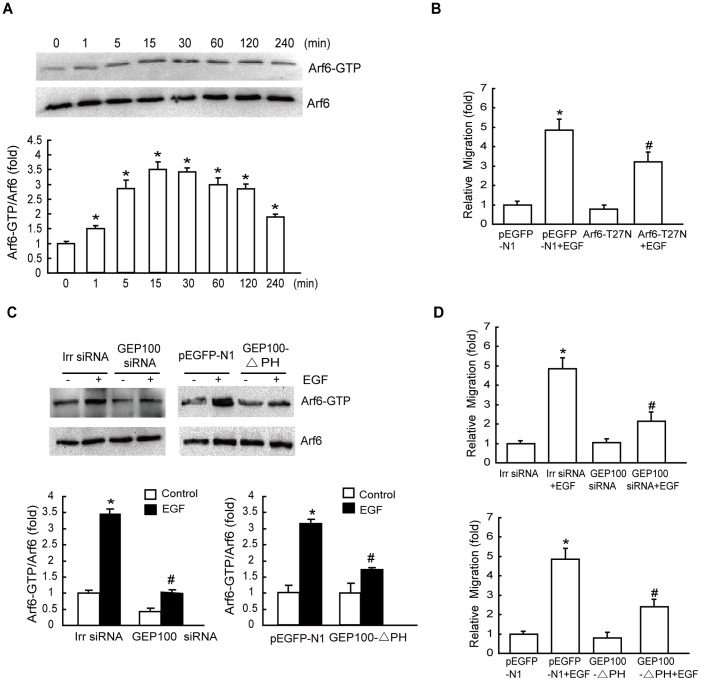
Effects of GEP100/Arf6 on EGF-induced cell migration. (A) EGF induces activation of Arf6. Serum-starved cell monolayers were treated with 10 ng/mL EGF for the indicated times. Cellular lysates were assayed for active Arf6 by pulldown assays as described in ‘Materials and methods’. The data were mean ± SD of three independent experiments. (B) Effect of the inactive mutant of Arf6 on EGF-stimulated migration. HepG2 cells were transiently transfected with the empty vector pEGFP-N1 and Arf6-T27N, respectively. Cells were then subjected to a Transwell migration assay in the presence of 10 ng/mL EGF for 8 h. (C) Both GEP100-siRNA and GEP100-△PH transfection inhibit Arf6 activation. Cells transfected with GEP100-siRNA or GEP100-△PH were stimulated with EGF for 15 min, and Arf6-GTP levels were examined. (D) Effects of the GEP100-siRNA and GEP100-△PH on EGF-stimulated cell migration. HepG2 cells infected with GEP100-siRNA and GEP100-△PH as indicated were subjected to a Transwell migration assay in the presence of 10 ng/mL EGF for 8 h. *: *P*<0.05, referring to the difference between cells treated with and without EGF. ^#^: *P*<0.05 (t-test), referring to the difference between the cells transfected with Arf6–T27N or GEP100 siRNA or GEP100-△PH plus EGF and the cells transfected with scrambled siRNA (irr siRNA) or empty vector plus EGF.

Accumulating evidence has indicated that Arf6 can be activated by various stress stimuli such as EGF [20]. We wished to examine whether Arf6 influenced cell migration in hepatoma cells after EGF treatment. We first investigated whether EGF could regulate Arf6 activation. Pulldown assays revealed that EGF induced Arf6 activation (Arf6-GTP) with an early peak at 15 min, which then returned to the basal levels, while the level of Arf6 protein in HepG2 cells remained unmodified during 4 h of EGF treatment (Fig. 2A). To determine whether EGF-induced cell migration was Arf6-dependent, we blocked Arf6 activity by transfecting these cells with Arf6 T27N (dominant negative mutant) (Fig. S1), and examined cell migration after EGF stimulation. We found that, in cells transfected with the empty vector, cell migration rate was increased significantly after the addition of EGF. However, in cells transfected with the Arf6 T27N expression vector, such stimulatory effect of EGF on cell migration was eliminated (Fig. 2B). These findings indicate that the activation of Arf6 is essential for EGF-stimulated hepatoma cell migration.

High level of GEP100, one of GEFs for Arf6, was expressed in HepG2 cells (Fig. S2). To determine whether EGF-stimulated Arf6 activity was GEP100-dependent, we blocked GEP100 expression by transfecting these cells with GEP100 siRNA (Fig. S3), and then examined Arf6 activity after EGF stimulation. We found that, compared with scrambled siRNA (irrelated), siRNA against GEP100 effectively reduced Arf6 activation in HepG2 cells after EGF-stimulation (Fig. 2C). Additionally, we examined the function of PH domain of GEP100 on EGF-induced Arf6 activation. The results revealed that transfection of GEP100-△PH markedly decreased Arf6 activation after stimulation with EGF. Expression levels of empty vector or GEP100-△PH were verified using total protein from HepG2 cells and immunoblotted using anti-GFP antibody (Fig. S4).

To further determine whether EGF stimulated cancer cell migration in a GEP100-dependent manner, we transfected HepG2 cells with GEP100 siRNA or GEP100-△PH and examined how these cells responded to EGF by Transwell migration assays. GEP100 siRNA or the mutant GEP100-△PH resulted in a remarkable inhibition of EGF-promoted cell migration (Fig. 2D), suggesting that GEP100, particularly its PH domain, is required for EGF-induced migration of these cells.

### GEP100/Arf6 Regulates ERK Activation during EGF-induced Cell Migration

**Figure 3:**
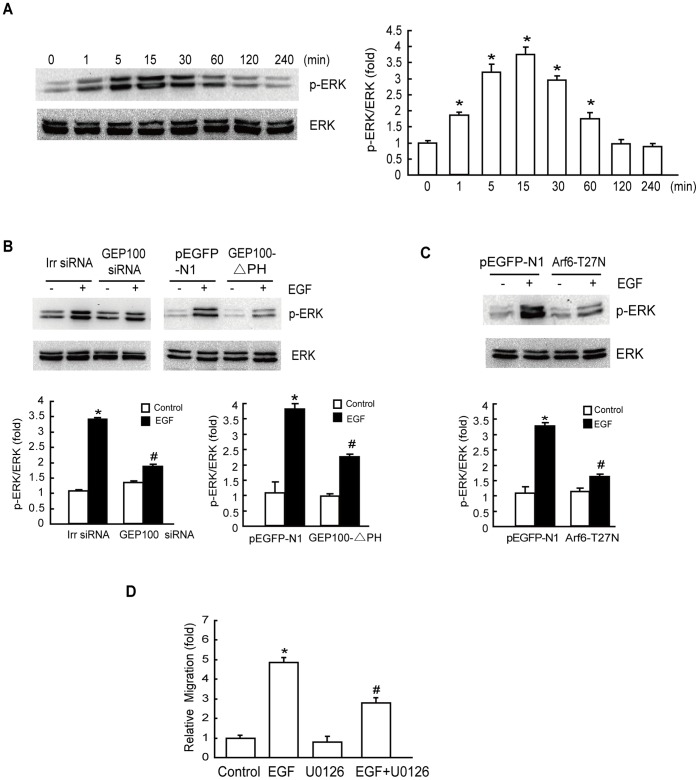
EGF activates ERK via the GEP100/Arf6 pathway that is required for EGF-induced cell migration. (A) Effect of EGF on the activation of ERK. HepG2 cells were starved overnight, followed by treatment with 10 ng/mL EGF for the indicated times. Phosphorylation of ERK at Thr202/Tyr204 was determined as described in ‘Materials and methods’. (B) Both GEP100-siRNA and GEP100-△PH transfection inhibit ERK activation. Cells transfected with GEP100-siRNA or GEP100-△PH were stimulated with EGF for 15 min, and the activation of ERK was examined. (C) EGF-activated ERK depends on Arf6. HepG2 cells were transiently transfected with the empty plasmid pEGFP-N1 and Arf6-T27N, respectively. The cells were then treated with or without EGF (10 ng/mL) for 15 min after an overnight serum starvation and ERK activity was examined. (D) Effect of ERK inhibitor on EGF-stimulated cell migration. After pretreatment with 10 µM U0126 for 60 min, HepG2 cells were incubated with 10 ng/mL EGF for 8 h and the cell migration rate was determined by Transwell migration assay. *: *P*<0.05, referring to the difference between cells treated with and those without EGF. ^#^: *P*<0.05 (t-test), referring to the difference between the cells transfected with Arf6–T27N or GEP100 siRNA or GEP100-△PH plus EGF and the cells transfected with scrambled siRNA (irr siRNA) or empty vector plus EGF.

To determine whether the regulation of HepG2 cell migration by GEP100 depended on ERK activation, we investigated the effect of GEP100/Arf6 on ERK activation using immunoblotting assays. We found that the level of phospho-ERK was significantly increased at 15 min after EGF stimulation, whereas the total protein level of ERK remained unaltered (Fig. 3A). The results showed that both GEP100 siRNA and GEP100-△PH largely inhibited EGF-induced ERK activity (Fig. 3B). ERK activity was also markedly inhibited by Arf6-T27N transfection (Fig. 3C). These results suggest that GEP100/Arf6 acts as an upstream effector of ERK activation.

The effect of ERK inhibitor on cell migration was also investigated. Pretreatment with 10 µM U0126 resulted in a remarkable inhibition of EGF-promoted cell migration (Fig. 3D). These results suggest that ERK acts as a downstream effector of GEP100 and Arf6 in mediating EGF-stimulated hepatoma cell migration.

### GEP100/Arf6 Regulates Rac1 Activation during EGF-induced Cell Migration

**Figure 4:**
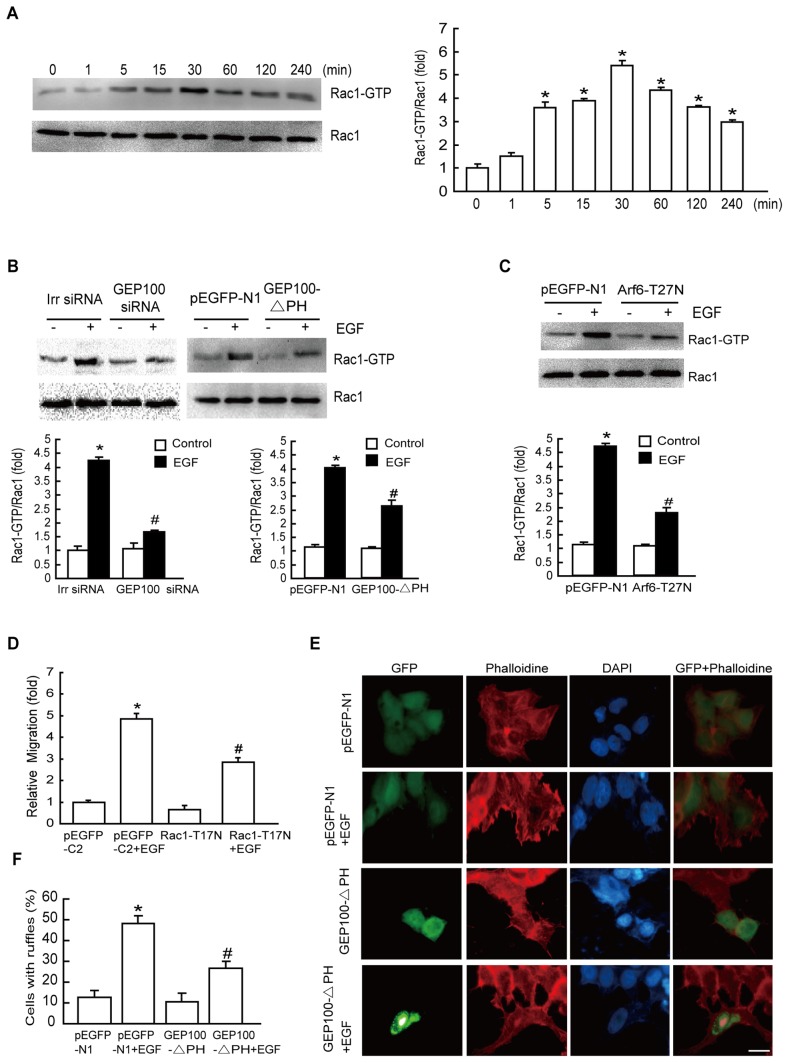
EGF activates Rac1 via the GEP100/Arf6 pathway that is required for EGF-induced cell migration. (A) Effect of EGF on the activation of Rac1. HepG2 cells were starved overnight, followed by treatment with 10 ng/mL EGF for the indicated times. Rac1 activation was determined as described in ‘Materials and methods’. (B) Both GEP100-siRNA and GEP100-△PH transfection inhibit Rac1 activation. Cells transfected with GEP100-siRNA or GEP100-△PH were stimulated with EGF for 30 min, and Rac1 activation was examined. (C) EGF-induced activation of Rac1 was dependent on Arf6. Cells transfected with Arf6-T27N were stimulated with EGF for 30 min, and Rac1 activation was examined. (D) Effect of Rac1-T17N on EGF-stimulated cell migration. After transfection with the empty vector or Rac1-T17N, HepG2 cells were incubated with 10 ng/mL EGF for 8 h and the cell migration rate was determined by Transwell migration assay. (E-F) GEP100-△PH blocked EGF-stimulated membrane ruffling. Cells expressing empty vector or GEP100-△PH were stimulated with EGF for 15 min, fixed and stained for the distribution of actin using TRITC-conjugated phaolloidin (red). Cells were counterstained with DAPI (blue). Images are representative of at least 3 independent determinations. Magnification, ×400. Scale bar, 20 µm. *: *P*<0.05, referring to the difference between cells treated with and without EGF. ^#^: *P*<0.05 (t-test), referring to the difference between cells transfected with Rac1-T17N, Arf6–T27N or GEP100 siRNA or GEP100-△PH plus EGF and the cells transfected with scrambled siRNA (irr siRNA) or empty vector plus EGF.

Accumulating evidence has indicated that Rac1 is a downstream effector of Arf6 in normal cells [21], [22], to determine whether Rac1 was the downstream target of GEP100/Arf6 activation by EGF in our system, we examined endogenous Rac1 activation after EGF treatment by immunoblotting assays. The results revealed a time-dependent increase in Rac1 activity following EGF treatment. Rac1 activation was significantly induced from 5 to 240 min after EGF stimulation with a peak at 30 min (Fig. 4A). We transfected GEP100 siRNA or overexpressed GEP100-△PH or Arf6-T27N in cells and then examined Rac1 activity after EGF stimulation. The results showed that Rac1 activation was largely abolished in cells expressing GEP100 siRNA, GEP100-△PH and Arf6-T27N, respectively, indicating that the GEP100/Arf6 signaling pathway is essential for EGF-stimulated Rac1 activation (Fig. 4B, 4C).

To investigate whether Rac1 activation was required for EGF-stimulated migratory effects, we blocked Rac1 activation by transfecting cells with a domain negative mutant of Rac1-T17N. The results showed that cells with Rac1-T17N prior to EGF treatment significantly suppressed cell migration (Fig. 4D), suggesting that activation of Rac1 downstream of GEP100/Arf6 is necessary for EGF-stimulated cell migration. Expression levels of empty vector and Rac1-T17N were verified using total protein from cells and immunoblotted using anti-GFP antibody (Fig. S5).

Cell motility requires extensions of the plasma membrane driven by reorganization of the actin cytoskeleton [23], so we performed fluorescent phalloidin staining to investigate the distribution pattern of F-actin in HepG2 cells. The results revealed that GEP100-△PH inhibited the formation of membrane ruffles induced by EGF (Fig. 4E). Thus, the findings from the cell biological assay are consistent with the biochemical evidence that GEP100 activation is required for cell motility.

### Inhibition of ERK Activity Suppresses GEP100/Arf6-mediated Rac1 Activation

**Figure 5:**
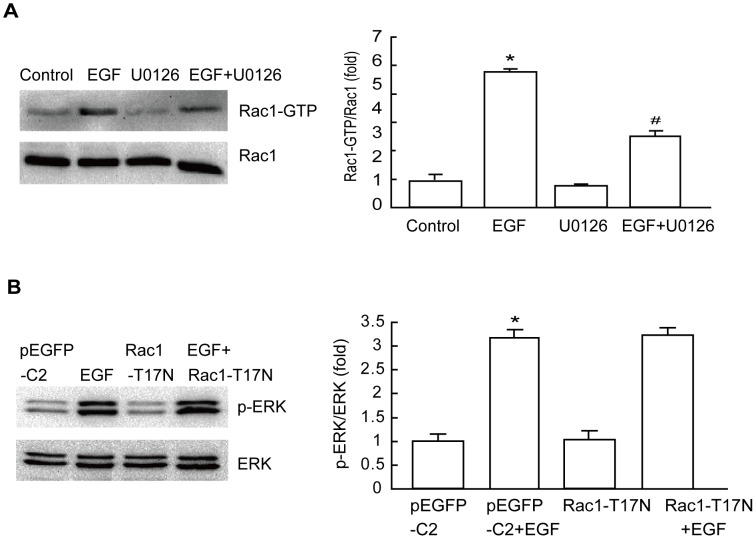
GEP100-dependent Rac1 activation requires ERK activity. (A) EGF-mediated activation of Rac1 requires ERK. HepG2 cells were treated with 10 µM U0126 for 60 min prior to EGF treatment (10 ng/mL) for 30 min, and then subjected to Rac1 activation analysis. (B) EGF-mediated ERK activation is Rac1 independent. Cells transfected with Rac1-T17N were stimulated with EGF for 15 min, and then subjected to ERK analysis. *: *P*<0.05, referring to the difference between cells treated with and those without EGF. ^#^: *P*<0.05 (t-test), referring to the difference between cells treated with EGF plus U0126 relative to cells treated with EGF alone.

ERK has been implicated in the Rac1 signaling pathway in various human cancer cell lines [24], [25], [26]. Therefore, we assessed whether ERK was also implicated in the EGF signaling pathway by analyzing activation of Rac1. The results showed that Rac1 activation was increased significantly in EGF-treated cells; however, the increases of these parameters were much less in the same treated cells pretreated with 10 µM U0126 (Fig. 5A). To determine whether the regulation between Rac1 and ERK in these cancer cells was bi-directional, cells transfected with Rac1-T17N were stimulated with EGF. We found that inhibition of Rac1 activation did not alter EGF-induced augmentation of ERK phosphorylation (Fig. 5B). These results suggest that ERK acts as an upstream molecule of Rac1 signaling.

## Discussion

Dysregulation of EGF and its receptors have been proved to promote tumor growth and metastasis of various types of cancer [6], [27]. In our system, HepG2 cancer cell migration rate was accelerated after EGF treatment compared with that of the control, but cell proliferation was not altered. Therefore, EGF directly activates the migration of hepatoma cells, which is a critical step for tumor metastasis. Based on this, the signaling mechanisms underlying the effect of EGF on promoting cancer cell migration were investigated.

A primary observation in the present study is that both GEP100 siRNA and PH domain deletion mutant GEP100-△PH transfection can significantly inhibit EGF-induced migration as well as membrane ruffling of HepG2 cells, indicating that GEP100, especially its PH domain, serves as a key mediator of EGF-stimulated migration of these cancer cells. It has been shown that the PH domain of GEP100 is associated with phosphorylated Tyr951 of VEGFR2 [28], and its PH domain was also reported to bind to certain phosphorylated tyrosines on EGFR, and mediate EGF-stimulated breast cancer cell invasion [14]. Similarly, our results not only showed that GEP100 is necessary for motility of HepG2 cancer cells *in vitro*, but also identified the effect of the PH domain of GEP100 in migration. Following transfection of HepG2 cells with GEP100-△PH plasmids, the migration of these cells was decreased significantly, showing that deletion of the PH domain of GEP100 in these cancer cells could impair the migration of these cancer cells *in vitro*.

GEP100 signaling is linked to the activation of Arf6 [29], [30]. Arf6 belongs to the Ras superfamily of GTP-binding proteins and its primary role is in membrane trafficking and structural organization at the plasma membrane [31]. There is consistent evidence that Arf6 can be activated by various growth factors, such as vascular growth factor [32], colony-stimulating factor [33], and G protein coupled receptor agonists [34]. Our results indicated that Arf6 plays an essential role in cancer cell migration during EGF stimulation. Like all GTPases, the Arf6 is under tight spatial control, which is mediated by guanine nucleotide exchange factors (GEFs) and GTPase-activating proteins (GAPs) that catalyse GTP exchange and hydrolysis, respectively. There are 15 genes in humans encoding proteins bearing the *Sec7* domain, a putative ArfGEF domain [35], [36]. Specific GEF allows Arf6 to be activated in specific signal transduction pathways and coordinate more elaborate responses to specific demands at localized cellular sites. Our results indicated that GEP100, the special GEF for Arf6, is responsible for EGF-induced Arf6 activation in HepG2 cells. Indeed, we show here that EGF-induced Arf6 activation could be suppressed by ectopic expression with GEP100 siRNA as well as GEP100-△PH, so we suggest that the PH domain of GEP100 is involved in EGF signaling to induce Arf6 activation and migration of human hepatoma HepG2 cells.

The ability of Arf6 to affect cortical actin cytoskeleton, cell shape and cell polarity is now well recognized [37]. A recent study has found that Arf6 is required for EGF-induced glioblastoma cell proliferation via the activation of PI3K and ERK signaling [38]. ERK has also been implicated in Arf6-mediated epithelial tubule development in response to hepatocyte growth factor (HGF) [39]. On the other hand, direct evidence that Arf6-GTP leads to Rac1 activation has been obtained [40]. Consistent with these reports, our results revealed that EGF-induced cell migration was associated with an increase in ERK and Rac1 activity. Inhibition of ERK activity by U0126 or suppression of Rac1 activity by ectopic expression of inactive mutant form of Rac1 (Rac1-T17N) significantly prevents EGF-induced cell migration, suggesting that EGF-induced ERK and Rac1 activation was responsible for the migration of these cancer cells. Furthermore, transfection of GEP100-△PH, inhibition GEP100 or Arf6 activity by GEP100 siRNA or Arf6 T27N failed to facilitate EGF-induced ERK and Rac1 activation. Therefore, it may be reasonable to speculate that EGF-induced ERK and Rac1 activation and cell migration require the PH domain of GEP100 and are mediated through the GEP100/Arf6 pathways. It has been reported that the EGFR-GEP100-Arf6-AMAP1 signaling pathway is specific to breast cancer invasion and metastasis [41]. Meanwhile, our data supported the concept that EGF is capable of inducing ERK and Rac1 activation through the Arf6 pathway, and this process is associated with hepatoma cell migration where GEP100 was present.

In some cell types, ERK is a downstream target of the Rac1 signaling cascade, such as in rat basophilic leukemia mast cells, and inactivation of Rac1 was sufficient to suppress ERK activation induced by eotaxin [42]. However, in our study, blocking ERK activity significantly prevents EGF-induced Rac1 activation. Furthermore, specific downregulation of Rac1 signaling in HepG2 cells did not alter EGF-induced activation of ERK. Our result is confirmed by a study in intestinal epithelial cells IEC-6, showing that ERK promoted cell adherens junctions through the activation of Rac1 [24]. The different results gained by different groups may be due to the different cell systems used and receptor-coupled signaling in these studies.

Rac1 is well-known required for the progression and metastasis of cancer cells by mediating growth factor-induced motility and invasiveness [43], [44]. However, it remains unclear whether GEP100/Arf6 mediates Rac1 activation through other pathways in our system. Rac1 is regulated by its GEFs and GAPs. A recent study showed that the DOCK180/Elmo complex, a Rac1 GEF, has been responsible for upregulating Rac1 activity and leads to migration of MDCK cells induced by Arf6 activation [45]. In addition, Arf6 is also found to form complexes with Rac1 and IQGAP1 in glioma cells upon HGF stimulation, and IQGAP1 is essential for Arf6-induced Rac1 activation and cell migration [46]. The role remains to be determined of other signaling molecules that function downstream of Arf6 in controlling Rac1 activation and cell migration.

In summary, we presented the first direct evidence that the PH domain of GEP100 is essential for cancer cell migration *in vitro*. We also identified a signaling pathway implicated in EGF-induced hepatoma cell migration in which GEP100 was present. EGF treatment can activate the GEP100-dependent Arf6/ERK/Rac1 cascade in hepatoma cancer cells, which contributes to the migrative ability of these cells. These findings are of potential pathophysiological importance for understanding the integration of migration-related signaling and shed light on new therapeutic targets for cancer.

## Materials and Methods

### Cells and Plasmids

Human hepatoma cell line HepG2 was obtained from the American Type Culture Collection (ATCC, Manassas, VA) and maintained at 37°C in high glucose Dulbecco’s modified Eagle’s medium (DMEM) (Gibco,Grand Island, NY) supplemented with 10% fetal bovine serum (FBS) (Hyclone, Logan, UT), 100 units penicillin/mL, and 100 µg/mL streptomycin in a humidified atmosphere. Cells were grown on coverslips for fluorescence staining and on plastic dishes for protein extraction. Cells were made quiescent by serum starvation overnight followed by drug treatment.

pEGFP-C2 vector containing dominant negative Rac1-T17N insert was kindly provided by Dr. Shoshana Ravid (The Hebrew University, Jerusalem, Israel). Dr. Julie G. Donaldson (Laboratory of Cell Biology, NIH) generously provided the construct of Arf6-T27N. Cells were transfected with either pEGFP-C2 or pEGFP-C2 expressing Rac1-T17N or Arf6-T27N using Lipofectamine 2000 as instructed by the manufacturer (11668, Invitrogen, Carlsbad, CA, USA). Cells were allowed to grow for 24 to 48 h post transfection before treatment with EGF where indicated. The pEGFP-GEP100-WT and pEGFP-GEP100-△PH were kindly gifted from Dr. Akimasa Someya. Details of plasmids are available upon request. The sequence of small interfering RNA (siRNA) for GEP100 was 5′-GCGAGAGCUAAAGACCAAUTT-3′ and for the scrambled sequence 5′-UUCUCCGAACGUGUCACGUTT-3′ (GenePharma Co.,Shanghai, China). Cells were grown until approximately 60% confluent and then transfected with GEP100 siRNA or scrambled siRNA using Lipofectamine 2000 as instructed by the manufacturer.

### Wound Closure Assay in vitro

Cells were plated in a 96-well plate. Approximately 48 h later, when cells were 95∼100% confluent, cells were incubated overnight in DMEM supplemented with 0.1% (w/v) BSA. Wounding was performed by scraping through the cell monolayer with a 10-µL pipette tip. Medium and non-adherent cells were removed, and cells were washed twice with PBS, and new medium with or without EGF (236-EG, R&D systems, Inc., Minneapolis, MN, USA) coupled with various inhibitors was added. Cells were permitted to migrate into the area of clearing for 24 h. Wound closure was monitored by visual examination under inverted Nikon TS100 microscope with a 100× objective.

### Transwell Migration Assay

HepG2 cells in exponential growth were harvested, washed, and suspended in DMEM without FBS. Cells (5×10^4^) were seeded into polycarbonate membrane inserts (8 µm pore size) in 24-Transwell cell culture dishes. Cells were allowed to attach to the membrane for 30 min before the addition of inhibitors. The lower chamber was filled with 600 µL DMEM without FBS containing 10 ng/mL EGF as a chemoattractant. Cells were permitted to migrate for 8 h. After the incubation, stationary cells were removed from the upper surface of the membranes. The cells that had migrated to the lower surface were fixed and stained with 0.1% crystal violet. The number of stained cells was counted under an ocular microscope.

### Cell Proliferation Assays

Cells were cultured in the absence or presence of 10 ng/mL EGF for 24 or 48 h and the proliferation of the cells was analyzed by staining with propidium iodide and flow cytometry analyses as described previously [47]. For MTT assay, cells were incubated with 10 ng/mL EGF for 24 or 48 h, before each time point, 20 µL MTT solution was added to each well followed by incubation at 37°C for 4 h. After removal of the medium, 150 µL dimethylsulfoxide (DMSO) was added to each well. After gentle shaking, absorbance at 490 nm was measured by using a plate reader (ELx800, BioTek Instruments, Inc., Vermont, USA). The OD difference between cells treated with or without EGF was calculated as relative content (% of control) and expressed graphically.

### Immunoblotting Analysis

Cellular lysates and immunoblotting were performed as previously depicted [48]. The following antibodies were used: rabbit anti-GEP100 (G4798, Sigma, St. Louis, MO, USA), mouse anti-Arf6 (sc-7971, Santa Cruz Biotechnology, Santa Cruz, CA, USA), mouse anti-Rac1 (05-389, Millipore, Billerica, MA, USA), rabbit anti-ERK1/2 and anti-phospho-ERK1/2 (Thr202/Tyr204) (4695,4377,Cell Signaling Technology, Boston, MA, USA), and mouse anti-GAPDH antibody (MAB374,Chemicon, Temecula, CA, USA). Digital images of the immunoblots were obtained with a Chemidoc XRS and analyzed using the image analysis program Quantity One (Bio-Rad, Hercules, CA, USA).

### Actin Cytoskeleton Staining and Immunofluorescence

Cells were fixed in 3.7% paraformaldehyde in PBS for 20 min, permeabilized in 0.2% Triton X-100 and blocked in PBS containing 1% BSA for 1 h at room temperature. The cells were incubated with mouse anti-GFP antibody (sc-53882, Santa Cruz) for 2 h followed by incubation with FITC-conjugated anti-mouse antibody for 1 h at room temperature within a moist chamber. F-actin was stained with TRITC-labeled phalloidin (5 µg/mL) (P1951, Sigma) for 40 min at room temperature. Following wash with PBS, the coverslips were mounted on glass slides with DAPI Fluoromount G (0100-20, Southern Biotech, Birmingham, AL). Images were collected using a fluorescent microscope (Lecia DM2500, Wetzlar, Germany). The filter cubes L5 (Excitation 480 nm, Emission 527 nm, Dichroic 505 nm) and TX2 (Excitation 560 nm, Emission 645 nm, Dichroic 595 nm) were used for GFP and F-actin observation, respectively.

### Pulldown Assays

Rac1 and Arf6 activity were measured as previously depicted [45], [49]. In brief,?equal volumes of total cellular protein were incubated with GST-RBD for detection of active Rac1, or GST-GGA3 for detection of active Arf6 (gifts from James E Casanova, University of Virginia, VA) beads captured on MagneGST Glutathione Particles (Promega, Madison, WI) for 1 h at 4 °C. The particles were then washed three times with washing buffer containing 4.2 mM Na_2_HPO_4_, 2 mM KH_2_PO_4_, 280 mM NaCl, and 10 mM KCl (pH 7.2), resuspended in 2×SDS sample buffer and subjected to immunoblotting analysis by using a mouse anti-Rac1 antibody (Upstate Biotechnology, Lake Placid, NY) or a mouse anti-Arf6 antibody (Santa Cruz Biotechnology, Santa Cruz, CA).

### Statistical Analysis

Statistical analysis was carried out using the SPSS software. Student’s *t* test was used to analyze the differences between two groups. When comparisons between multiple groups were carried out, one-way ANOVA followed by SNK tests were employed. Statistical significance was considered at *P*<0.05.

## Supporting Information

Figure S1 **Expression of Arf6-T27N in HepG2 cells.** Expression levels of empty vector and Arf6-T27N were verified using total protein from cells and immunoblotted using anti-GFP antibody.
(TIF)

Figure S2 **Protein levels of GEP100 in HepG2 cells.** The level of GEP100 expression in HepG2 cells was determined as described in ‘Materials and methods’. MCF-7 cells were used as negative control. MDA-MB-231 cells were used as positive control.
(TIF)

Figure S3 **The effect of siRNA on the intracellular levels of GEP100.** Total protein extracts from HepG2 cells transfected with siRNA-GEP100 or scrambled siRNA (mock) were analyzed by Western blotting for GEP100. GAPDH was used as loading control.
(TIF)

Figure S4 **Expression of GEP100-△PH in HepG2 cells.** Expression levels of empty vector and GEP100-△PH were verified using total protein from cells and immunoblotted using anti-GFP antibody.
(TIF)

Figure S5 **Expression of Rac1-T17N in HepG2 cells.** Expression levels of empty vector and Rac1-T17N were verified using total protein from cells and immunoblotted using anti-GFP antibody.
(TIF)
