# Supplementary figures and images for: GEP100/Arf6 Is Required for Epidermal Growth Factor-Induced ERK/Rac1 Signaling and Cell Migration in Human Hepatoma HepG2 Cells
Source: PLoS One. 2012 Jun 11;7(6):e38777. doi: 10.1371/journal.pone.0038777 (PMC3372492; doi:10.1371/journal.pone.0038777)

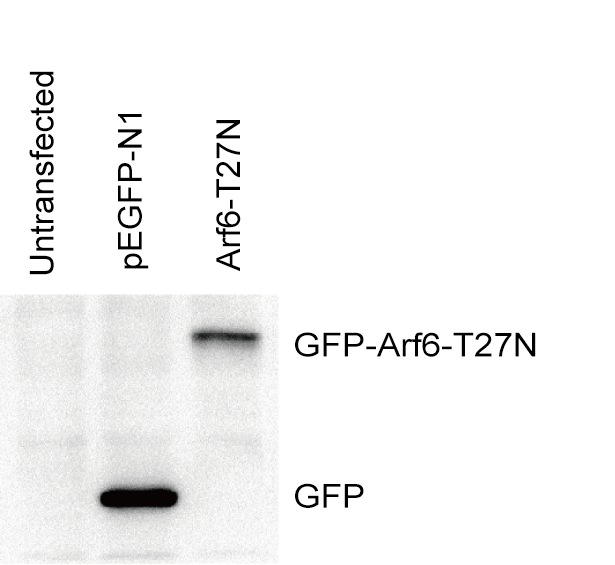

Supplement: Figure S1 — Expression of Arf6-T27N in HepG2 cells. Expression levels of empty vector and Arf6-T27N were verified using total protein from cells and immunoblotted using anti-GFP antibody. (TIF) [file pone.0038777.s001.tif]

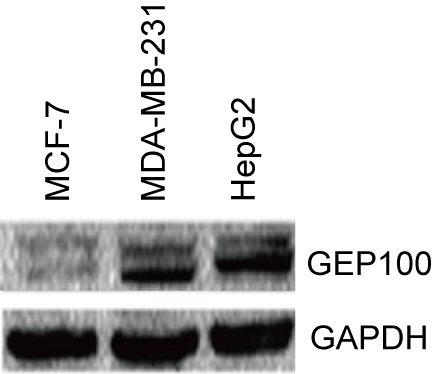

Supplement: Figure S2 — Protein levels of GEP100 in HepG2 cells. The level of GEP100 expression in HepG2 cells was determined as described in ‘Materials and methods’. MCF-7 cells were used as negative control. MDA-MB-231 cells were used as positive control. (TIF) [file pone.0038777.s002.tif]

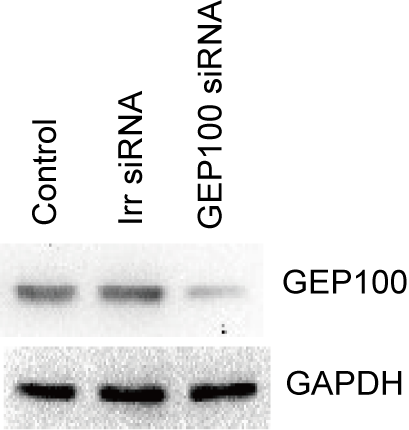

Supplement: Figure S3 — The effect of siRNA on the intracellular levels of GEP100. Total protein extracts from HepG2 cells transfected with siRNA-GEP100 or scrambled siRNA (mock) were analyzed by Western blotting for GEP100. GAPDH was used as loading control. (TIF) [file pone.0038777.s003.tif]

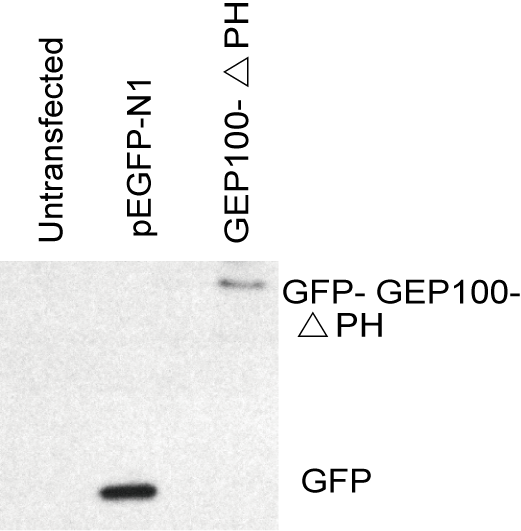

Supplement: Figure S4 — Expression of GEP100-△PH in HepG2 cells. Expression levels of empty vector and GEP100-△PH were verified using total protein from cells and immunoblotted using anti-GFP antibody. (TIF) [file pone.0038777.s004.tif]

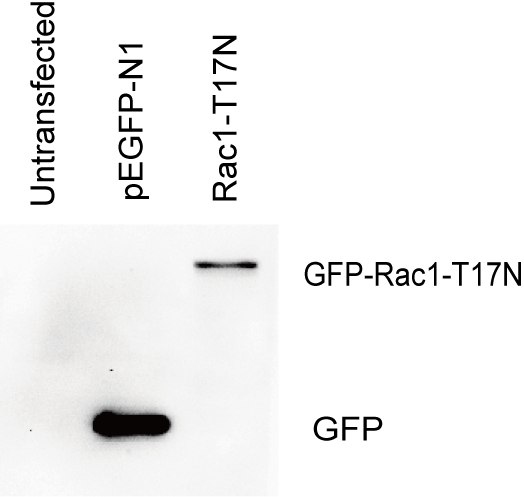

Supplement: Figure S5 — Expression of Rac1-T17N in HepG2 cells. Expression levels of empty vector and Rac1-T17N were verified using total protein from cells and immunoblotted using anti-GFP antibody. (TIF) [file pone.0038777.s005.tif]
